# Real-Time Driving Behavior Identification Based on Multi-Source Data Fusion

**DOI:** 10.3390/ijerph19010348

**Published:** 2021-12-29

**Authors:** Yongfeng Ma, Zhuopeng Xie, Shuyan Chen, Ying Wu, Fengxiang Qiao

**Affiliations:** 1 Jiangsu Key Laboratory of Urban ITS, School of Transportation, Southeast University, Nanjing 211189, China; 220203282@seu.edu.cn (Z.X.); 220203386@seu.edu.cn (Y.W.); 2 Innovative Transportation Research Institute, Texas Southern University, Houston, TX 77004, USA; fengxiang.qiao@tsu.edu

**Keywords:** real-time driving behavior identification, stacked long short-term memory network, data fusion, time window, online car-hailing, driver expression data

## Abstract

Real-time driving behavior identification has a wide range of applications in monitoring driver states and predicting driving risks. In contrast to the traditional approaches that were mostly based on a single data source with poor identification capabilities, this paper innovatively integrates driver expression into driving behavior identification. First, 12-day online car-hailing driving data were collected in a non-intrusive manner. Then, with vehicle kinematic data and driver expression data as inputs, a stacked Long Short-Term Memory (S-LSTM) network was constructed to identify five kinds of driving behaviors, namely, lane keeping, acceleration, deceleration, turning, and lane changing. The Artificial Neural Network (ANN) and XGBoost algorithms were also employed as a comparison. Additionally, ten sliding time windows of different lengths were introduced to generate driving behavior identification samples. The results show that, using all sources of data yields better results than using the kinematic data only, with the average F1 value improved by 0.041, while the S-LSTM algorithm is better than the ANN and XGBoost algorithms. Furthermore, the optimal time window length is 3.5 s, with an average F1 of 0.877. This study provides an effective method for real-time driving behavior identification, and thereby supports the driving pattern analysis and Advanced Driving Assistance System.

## 1. Introduction

With the increasing demands of road traffic safety and the decreasing cost of data acquisition equipment, driving behavior identification has become a research hotspot in recent years. Real-time driving behavior identification is one of the most important basic modules of driving assistance systems [1,2], which has a wide range of applications in driver state monitoring [3,4,5], driving style analysis [6,7,8,9], automobile insurance [1], and fuel consumption optimization [10].

Conceptually, there are two types of driving behaviors: macroscopic driving behavior and microscopic driving behavior. The macroscopic driving behavior refers to the overall driving states or patterns of the vehicle during a relatively long period of driving, such as normal, fatigue, aggressive, and other stages [11,12], while the microscopic driving behavior is more specific to short-range operations, usually including stop, acceleration, deceleration, and others, and is the focus of this paper. Referring to previous studies [13,14,15,16,17], the five most typical driving behaviors were selected in this paper: lane keeping, acceleration, deceleration, turning, and lane change.

Currently, the methods of driving behavior identification can be divided into two main categories. One uses the thresholds of vehicle kinematic parameters to identify the start and end of driving behaviors. Ma et al. [18] identified three kinds of driving behaviors, namely, turning, acceleration, and deceleration, by thresholds of vehicle angular velocity and longitudinal acceleration, with three styles, aggressive, normal, and cautious, to explore the differences in driving styles during different driving stages of online car-hailing. Although this method is simple and easy to comprehend, most of the thresholds in existing research are determined empirically, leading to weak identification effects, with the identified driving behaviors requiring manual verifications in most cases. Recently, the machine learning and deep learning-based methods have become more popular in transportation studies. Since the driving behaviors usually last for a period of time, identification is essentially a time series-based classification problem, where each sample is a two-dimensional matrix, including a time dimension and a feature dimension. Classic machine learning algorithms usually solve such problems by flattening features or dividing the time series into different time windows to extract the statistical features, such as mean, standard deviation, or other metrics, as input [19,20]. However, these extracted features could not reflect the chronological features of driving behaviors; thus, some researchers used the dynamic time warping (DTW) algorithm to reflect the sequence-to-sequence distance more reasonably [14]. Zheng et al. [1] used the KNN-DTW algorithm to identify two kinds of driving behaviors: left lane change and right lane change, with an identification accuracy of 81.97% and 71.77%, respectively. Considering the fact that it was a simple binary classification problem, such accuracies were relatively low, which can be attributed to the weakness of the algorithm itself. A more efficient method is to use deep learning algorithms to improve the nonlinearity and generalization ability [21]. Xie et al. [22] used the UAH-DriveSet public dataset, selected seven features, namely, velocity, tri-axis accelerations, and tri-axis angular velocities, and employed a convolutional neural network (CNN) to identify six kinds of driving behavior: lane keeping, acceleration, braking, turning, left lane change, and right lane change, with an average F1 of 0.87. Similarly, Xie et al. [16] compared three window-based feature extraction methods, statistical values, principal component analysis, and stacked sparse auto-encoders, and used the random forest method to classify driving behaviors on three public datasets: UAH, UW, and IMS.

The data used in the above research were all vehicle kinematic data only, and the limited information from a single source leads to poorer identification results. Driving behavior is not only closely related to vehicle motion, it is also expressed through driver states. Therefore, some researchers have incorporated driver states, such as eye-movement features [23] and physiological features [24], to analyze driving behaviors. Guo et al. [25] identified the driving intention to lane change using a bidirectional LSTM network based on an attention mechanism with vehicle kinematic data, driver maneuver data, driver eye-movement data, and head rotation data as input, and achieved an accuracy of 93.33% at 3 s prior to the lane change. However, the eye-movement data and physiological data are mostly collected in an intrusive manner, which can cause some interference with driving. To the authors’ knowledge, there is currently no research on real-time driving behavior identification considering drivers’ facial expressions. Fortunately, it has been shown that drivers’ driving behaviors and emotions are closely related [26,27,28]. A study by Precht et al. [29] showed that drivers’ anger emotions can increase the frequency of their aggressive driving behaviors, such as rapid deceleration and rapid turning, while such aggressive driving behaviors are intentional rather than potentially influenced by emotions. Kadoya et al. [30] found that negative emotions (angry and sad) of taxi drivers had significant impacts on increased driving speed, while a neutral emotional state is related to decreased speed. It is common sense to use driver expressions to identify driving behaviors. For example, drivers need to look at both side-mirrors when turning or changing lanes, which may lead to changes in expressions (such as eyebrow shapes), and in fact the eye-movement features in some studies were also deduced from driver expressions [31].

In order to integrate driver expressions into driving behavior identification without interfering with drivers and to monitor vehicle movement and driver states in real time for better analyzing driving style and risks, this study conducts an online car-hailing naturalistic driving test to collect vehicle kinematic data and driver expression data in a non-intrusive way and uses the sliding time window on the whole data set to generate driving behavior identification samples. The stacked long short-term memory (S-LSTM) network is employed to identify five kinds of driving behaviors, namely, lane keeping, acceleration, deceleration, turning, and lane change, while the artificial neural network (ANN) and XGBoost algorithms are used as a comparison to validate the effectiveness of the S-LSTM algorithm.

## 2. Data Collection and Pre-Processing

An online car-hailing naturalistic driving test was conducted in Nanjing, China, where drivers drove in real scenarios without any test-related interference. By posting recruitment information in the Nanjing online car-hailing platform, a total of 22 drivers were recruited. However, there were 10 drivers whose faces were occluded or far away from the camera, and the expression data could not be well recognized, thus a total of 12 drivers were finally employed. Considering the long test time and high sampling frequency, a dataset with the test results from 12 drivers should already be sufficient [32,33,34]. Due to the specificity of this occupation, the 12 drivers were all male, with an average age of 36 years old, and they all had three or more years’ driving experience. These drivers were informed of the specific requirements of the test and received sufficient training before the formal test. The test was conducted during the daytime (08:00–20:00) under good weather conditions. In order to ensure the generality of the road environment, the test vehicles were free to take orders in the Nanjing area, with no restrictions on the test routes, including urban expressways, major arterial roads, minor arterial roads, and local streets. The data collection during the test was made in a non-intrusive way so as to ensure the validity of the data.

**Figure 1:**
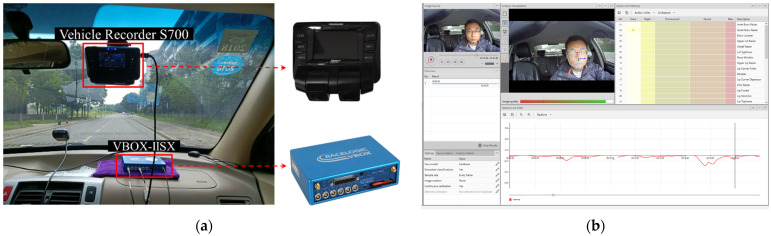
Test equipment. (**a**) In-vehicle equipment, (**b**) FaceReader 8.0.

In this test, the vehicle kinematic data were collected by VBOX-IISX GPS data loggers with a sampling frequency of 10 Hz. The outside ambient video and driver video information were collected by the S700 Vehicle Recorder, while the driver expression data were obtained through the processing of the driver videos with the FaceReader 8.0, a tool that automatically analyses facial expressions and provides an objective assessment of a driver’s motion. The test equipment is shown in Figure 1.

The data were collected over 12 days from the end of 2018 to early 2019 and consisted of 3,120,578 pieces of vehicle kinematic data and 905.5 GB of video clips. The vehicle kinematic data contained 12 features, which are described in Table 1. The video clip data include the information from outside ambient videos and driver videos.

Driver expression data were obtained by processing driver videos using FaceReader 8.0, including the action intensity of the 20 most common facial action units with a frequency of 10 Hz and a value range of 0–1, where larger values indicate greater action intensity. The description of each action unit is shown in Table 2.

Due to the equipment problems, both vehicle kinematic data and driver expression data had a small portion of missing values. As the data sampling frequency is relatively high (10 Hz), a linear interpolation could be sufficient to successfully interpolate those missing data.

Yaw rate plays an important role in driving behavior analysis [14,15,16], which was then calculated from the heading information. First, the heading data was converted to the cumulative heading angle for expansion to the arbitrary angle. Then, the cumulative heading angle was differentiated to obtain the yaw rate with the unit of (°/s).

In addition, to reduce the noise and improve the stability, the vehicle kinematic data were smoothed using a Savitzky–Golay filter, and the specific steps and parameters were taken from a study by Brombacher et al. [6].

## 3. Methodology Description

The notation and description for used variables are shown in Table 3.

### 3.1. Feature Selection

**Figure 2:**
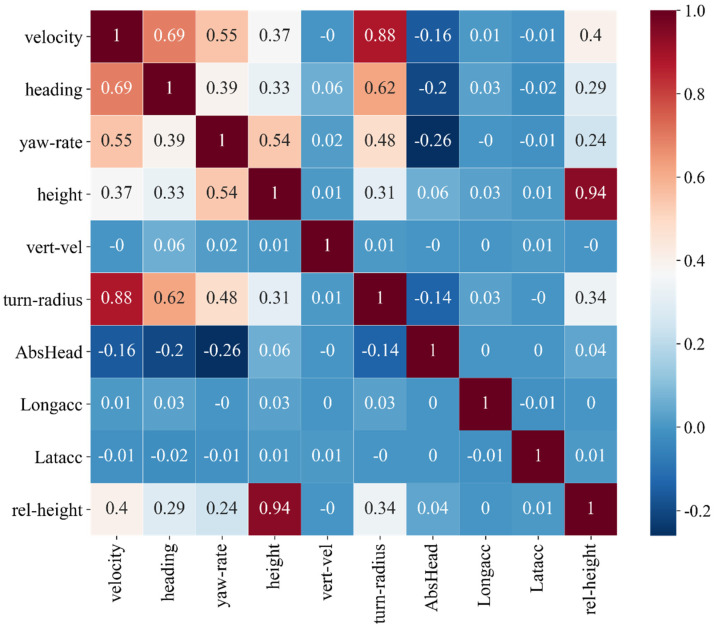
Correlation analysis of vehicle kinematic data.

The correlation analysis was performed on a partial of features of the vehicle kinematic data, with the results shown in Figure 2. The correlation coefficient between Height and Rel-height is 0.94, which is highly positively correlated, and the feature Height is retained here. The correlation coefficient between Velocity and Turn-radius is 0.88, indicating a high and positive linear correlation, and the feature Velocity is retained also. In addition, several other features that contribute little to the identification efforts were eliminated. Finally, seven features were selected: (1) Velocity, (2) Heading, (3) Yaw rate, (4) Height, (5) Vertical speed (Vert-vel), (6) Longitude acceleration (Longacc), and (7) Latitude acceleration (Latacc). Twenty action units of driver expression data are all typical human facial features, so all of them were retained. Therefore, the entire driving behavior identification dataset contains 27 features.

### 3.2. Driving Behavior Labeling and Sliding Time Window

This study identifies five kinds of driving behaviors: lane keeping, acceleration, deceleration, turning, and lane change. To improve the accuracy and the efficiency, these five driving behaviors were labeled based on a combined consideration of the outside ambient video information and the thresholds of the vehicle kinematic data. The specific labeling steps are as follows.

**Figure 3:**
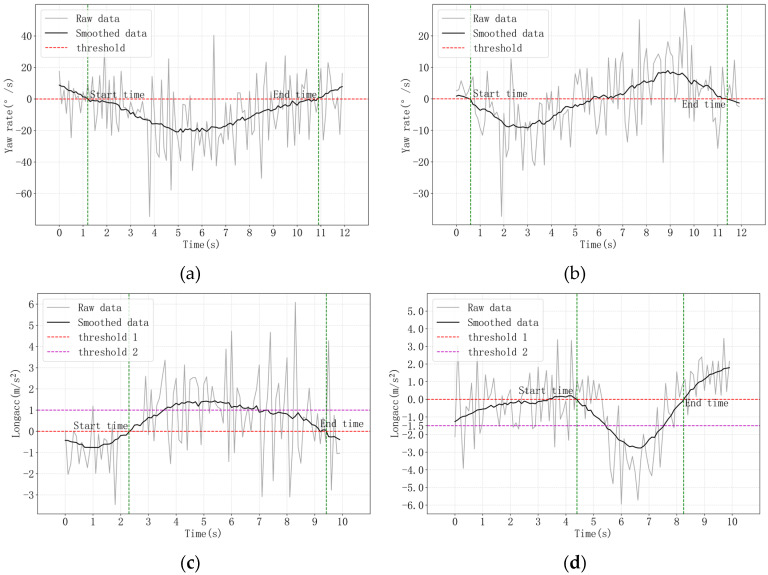
The principle of driving behaviors labeled by thresholds. (**a**) Turning behavior, (**b**) Lane-change behavior, (**c**) Acceleration behavior, (**d**) Deceleration behavior.

(1) For turning and lane-change behavior, the approximate start and end time were first labeled through the outside ambient videos, and then the extracted segments were labeled through the thresholds of the yaw rate. Specifically, the sign of the yaw rate is always the same when turning, while the yaw-rate line for lane-change behavior will cross the *x*-axis, as shown in Figure 3a,b, respectively.

(2) For acceleration and deceleration behavior, the identification thresholds were set according to previous studies [4,14]. The time period where Longacc≥0, $t_{d}\geq 1\;s$ and $Longacc_{\max}\geq 1\;{m/s}^{2}$, as well as the period where Longacc≤0, $t_{d}\geq 1\;s$ and $Longacc_{\min}\leq -1.5\;{m/s}^{2}$ were extracted first, are shown in Figure 3c,d, respectively. Then, the segments where the vehicle is in a straight state were further extracted by watching the outside ambient videos.

(3) For lane keeping behavior, combining previous studies [35,36,37] and the specific situation of this study, the period where |yaw rate|≤5 °/s was extracted first, then the extracted segments were further verified by outside ambient videos.

After the above steps, some unlabeled frames (0.1 s), defined by other behaviors [13], will be labeled in subsequent processes. Furthermore, it should be noted that acceleration and deceleration may be included in the process of turning and lane change, so the labeling priority was defined as: the priority for turning and lane change > acceleration and deceleration > lane keeping. This means, the overlapped frames were labeled with the driving behaviors with higher priorities.

Through the above four steps, a total of 1789 lane-keeping behaviors, 1676 acceleration behaviors, 1885 deceleration behaviors, 1020 turning behaviors, and 1436 lane-change behaviors were labeled. The label indices of driving behaviors are defined as: 1 for lane keeping, 2 for acceleration, 3 for deceleration, 4 for turning, and 5 for lane change.

In order to meet the real-time requirements, it was necessary to introduce the sliding time window to identify driving behaviors using the vehicle kinematic and expression data in the past period of time. The sliding interval of the time window was exactly the frequency of the identification of driving behavior. In this study, the sliding interval was set to 1 s for real-time performance. The length of the time window was referred to previous studies [16,38]: l∈{0.5 s, 1 s, 1.5 s, 2 s, 2.5 s , 3 s , 3.5 s, 4 s, 4.5 s , 5 s}, and the optimal length would be explored through subsequent experiments. Each time window was labeled using Algorithm 1 [23]. After video confirmation, it was found that the majority of other behaviors were performed when the vehicles were parked, and a small number of data were missing. Since other behaviors are not within the scope of this study, they will not be considered in the subsequent analysis.
**Algorithm 1:** Label generation for the dataset.**Input**: The label of each frame of the dataset**Output**: Label of each time window, where 0: other behaviors, 1: lane keeping, 2: acceleration, 3: deceleration, 4: turning, 5: lane change**For each time window:****if** all frames in the time window are unlabeled **then**Set 0 as the label of the time window**else**Compute the mode of the labels in the time windowSet the mode as the label of the time window**End for**

### 3.3. Driving Behavior Identification Models

After determining the time windows and the corresponding labels, the driving behavior identification is essentially a multidimensional time series-based classification problem. The S-LSTM network is constructed, which is a common time series algorithm in deep learning, while the ANN and XGBoost algorithms are used for the verification of the effectiveness of S-LSTM.

#### 3.3.1. Input Data

Each input sample of the S-LSTM model is a two-dimensional matrix containing a feature dimension and a time dimension. The feature dimension consists of a total of 27 vehicle kinematic and driver expression features, while the time dimension is a time series with a time interval of 0.1 s. Therefore, the shape of each input sample is 27∗10l. For the ANN and the XGBoost, four statistic variables, namely, minimum, maximum, mean, and standard deviation, were calculated for the 27 features under each time window, thus each input sample is a one-dimensional vector with the length 27∗4=108.

#### 3.3.2. Model Structure and Parameters

**Figure 4:**
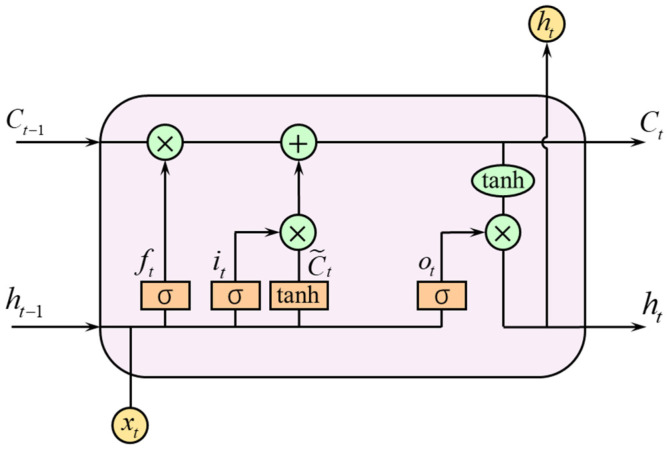
Unit structure of LSTM [40].

The LSTM network was first proposed by Hochreiter et al. [39] in 1997 and is a variant of the recurrent neural network (RNN), delivering good solutions to problems of slow training of RNN, which is beneficial in dealing with time series problems. The neural unit of LSTM has a gate structure, including the forgetting gate, input gate, and output gate, as shown in Figure 4. The computation process of each neural unit is shown in Equations (1)–(6).

(1) $$f_{t}=\sigma (W_{f}\cdot \left[h_{t-1},x_{t}\right]+b_{f})$$

(2) $$i_{t}=\sigma (W_{i}\cdot \left[h_{t-1},x_{t}\right]+b_{i})$$

(3) $${\tilde{C}}_{t}=\tanh(W_{C}\cdot \left[h_{t-1},x_{t}\right]+b_{C})$$

(4) $$C_{t}=f_{t}\cdot C_{t-1}+i_{t}\cdot {\tilde{C}}_{t}$$

(5) $$o_{t}=\sigma (W_{o}\cdot \left[h_{t-1},x_{t}\right]+b_{o})$$

(6) $$h_{t}=o_{t}\cdot \tanh(C_{t})$$

where, $f_{t}$, $i_{t}$, $o_{t}$ are the outputs of the forgetting gate, input gate, and output gate at the current moment, respectively; $C_{t-1}$, $C_{t}$ are the unit states of the previous moment and the current moment, respectively; ${\tilde{C}}_{t}$ is the candidate value of $C_{t}$; $h_{t-1}$ is the output of the hidden layer at the previous moment; $x_{t}$ is the input at the current moment; $W_{f}$, $W_{i}$, $W_{C}$, $W_{o}$ are the weight matrices; $b_{f}$, $b_{i}$, $b_{C}$, $b_{o}$ are the bias vectors; and σ is a sigmoid function.

**Figure 5:**
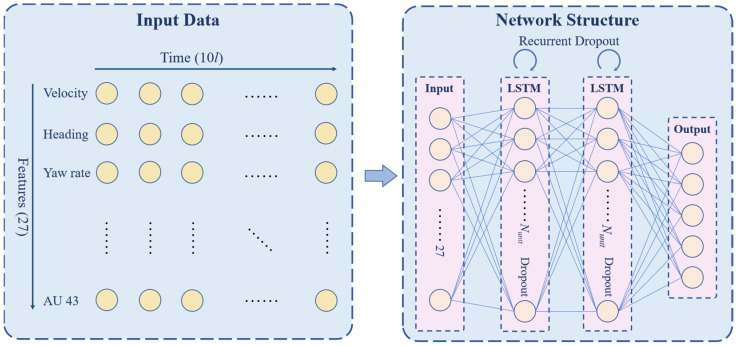
The input data and structure of the network.

Due to the large amount of data, the S-LSTM was constructed using recurrent layer stacking technology to improve the learning ability of the model, as shown in Figure 5. The network consists of an input layer, two LSTM layers, and an output layer. The activation functions of the LSTM layers and the output layer are ReLU and Softmax, respectively. In order to prevent overfitting, both Dropout and Recurrent Dropout regularization were used in LSTM layers. Early-stopping was adopted during the training, the validation loss was selected as the monitor, and the patience was set to 3.

The data set was divided into a training set and a test set at a ratio of 8:2, and the parameters of the S-LSTM, ANN, and XGBoost were adjusted, respectively, using a five-fold cross-validation, while the optimal parameters were finally determined as shown in Table 4.

### 3.4. Model Evaluation

Four measurement metrics, namely, accuracy, precision, recall, and F1, were selected to evaluate the candidate models, while the calculation formulas are shown in Equations (7)–(10). Besides the macro-average precision, recall, and F1, the arithmetic average values of precision, recall, and F1 of each driving behavior are selected to evaluate the overall effects of the models.

(7) $$Accuracy=\frac{TP+TN}{TP+FP+FN+TN}$$

(8) $$Precision=\frac{TP}{TP+FP}$$

(9) $$Recall=\frac{TP}{TP+FN}$$

(10) $$F1=\frac{2\cdot Precision\cdot Recall}{Precision+Recall}$$

where, TP, FP, FN, and TN are the number of true positive, false positive, false negative, and true negative results, respectively.

In addition, a receiver operating characteristic (ROC) curve is also used to visualize model performance, the horizontal coordinate of which is the false positive rate (FPR) (see Equation (11)), while the vertical coordinate is the true positive rate (TPR), namely, recall (see Equation (9)). The measurement variable area under the curve (AUC) is the total area under the ROC curve, while its larger value represents better classification results.

(11) $$FPR=\frac{FP}{FP+TN}$$

## 4. Results and Discussion

### 4.1. Comparison of Different Input Features

The identification results of the S-LSTM model using all data (ALL) and using the vehicle kinematic data only (VK) are shown in Table 5. The F1 of S-LSTM (ALL) for identifying lane keeping, acceleration, deceleration, and turning behaviors are all around 0.9, while the average F1 of the five types of driving behaviors is 0.877, which is 0.041 higher than that of S-LSTM (VK). These results indicate that driver expressions are correlated with driving behaviors, and the S-LSTM model is good in handling high-dimensional features and mining rich information from multi-source data.

### 4.2. Comparison with Different Algorithms

The identification results of the ANN and XGBoost for each driving behavior are shown in Table 6. Comparing Table 5 with Table 6, it can be found that the results of S-LSTM are significantly better than those of the ANN and the XGBoost algorithms. The results of the ANN are not so perfect, with an average F1 of 0.652, while the F1 for identifying lane-change behavior is only 0.542, which is close to a random guess. The difference in model performance may be attributed to the existence of a certain duration of driving behaviors, and the S-LSTM can better capture the chronological features, which could be lost when using the ANN and the XGBoost by extracting features through statistics.

**Figure 6:**
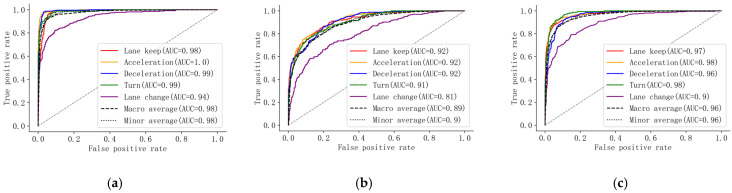
The ROC curve for each algorithm. (**a**) S-LSTM, (**b**) ANN, (**c**) XGBoost.

Figure 6 shows the ROC curves and AUCs of each algorithm to identify the different driving behaviors. It can be seen from Figure 6 that the ROC curves of the S-LSTM scenario for each driving behavior, macro-average, and micro-average are all closer to the upper left, and the AUCs are also significantly higher than those of ANN and XGBoost, indicating the superiority of the S-LSTM again. In addition, the ANN and the XGBoost have high AUCs for each driving behavior, although the overall identification results are not so perfect, indicating that these two algorithms can be competent for the binary classification problem. However, when the number of categories increases, they will confuse different kinds of driving behaviors.

### 4.3. Comparison of Different Time Windows

**Figure 7:**
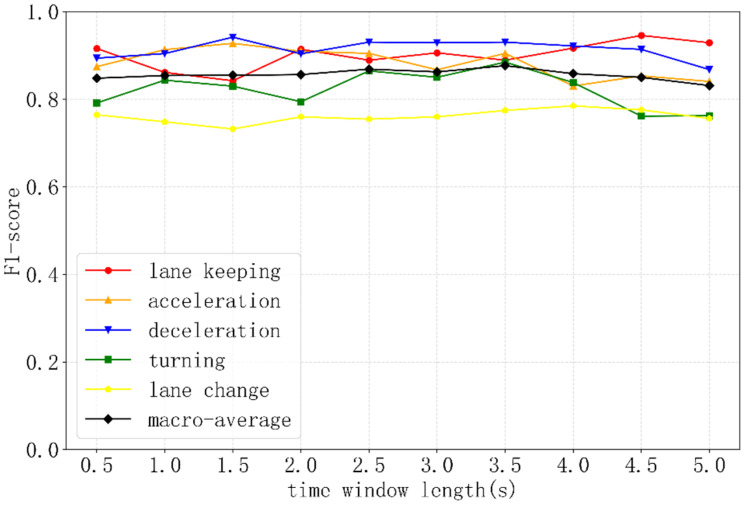
Identification results of different time windows.

For the ten time windows, namely, l∈{0.5 s, 1 s, 1.5 s, 2 s, 2.5 s , 3 s , 3.5 s, 4 s, 4.5 s , 5 s}, the F1 of the S-LSTM for identifying the five kinds of driving behaviors is shown in Figure 7. As can be seen from Figure 7, the acceleration and deceleration behaviors are well identified when the time window is short, while the turning and lane-change behavior results are better when the time window is relatively longer. The reason is that turning and lane-change behaviors usually have longer durations, so a short time window fails to capture the information completely. In contrast, acceleration and deceleration behaviors usually last for a short time period, so a longer time window tends to capture unnecessary information. Moreover, the identification results of lane-keeping behaviors fluctuate greatly with the length of the time windows because of the large difference in the duration of lane-keeping behaviors. Overall, the macro-average F1 of the five driving behaviors is stable around 0.8–0.9, and the results do not differ much between different time windows. In practical applications, the recommended time window length is 3.5 s, with an average F1 of 0.877, which is basically consistent with the previous study [16].

### 4.4. Comparison of Different Driving Behaviors

**Figure 8:**
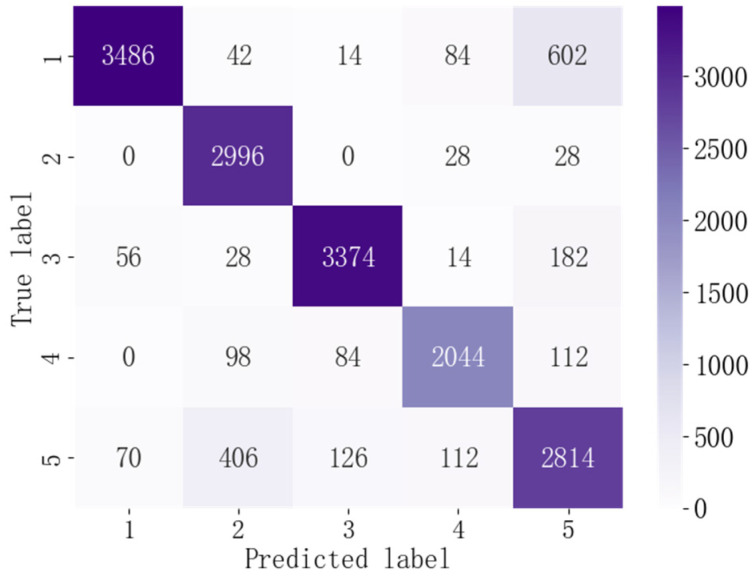
Confusion matrix of driving behavior identification.

Figure 8 shows the confusion matrix of the S-LSTM model with respect to identifying each driving behavior, where 1, 2, 3, 4, and 5 represent lane-keeping, acceleration, deceleration, turning, and lane-change behaviors, respectively. From Figure 8, it can be seen that the S-LSTM model provides relatively lower identification results for lane-change behaviors and misclassifies some lane-keeping behaviors as lane-change behaviors, which may be due to the fact that lane change usually contains short lane-keeping fragments that are easily confused. The results of lane-keeping, acceleration, deceleration, and turning behaviors are better, which can be attributed to the fact that these behaviors are relatively simple and the S-LSTM model can better capture their features.

## 5. Conclusions

In this paper, a non-intrusive method was used to collect high-precision vehicle kinematic data and high-definition video data through an online car-hailing naturalistic driving test in Nanjing, which ensured the capture of rich information on driving behaviors with no interference to driving, improving the identification of driving behaviors, and providing theoretical support for analyzing driving behavior using multi-source data.

In order to determine a reasonable time dimension, 10 different time windows were compared for generating driving behavior samples. The S-LSTM model was constructed to identify five kinds of driving behaviors: lane keeping, acceleration, deceleration, turning, and lane change, while two machine learning algorithms, the ANN and the XGBoost, were used as comparisons to verify the effectiveness of the S-LSTM model. The results show that the identification effect of the S-LSTM is better than the ANN and the XGBoost algorithms, the driver expression data enhancing the identification results, which implies that the S-LSTM model could mine richer information from multi-source data and is suitable for solving such time series classification problems with many features and large data volumes. It has also been demonstrated that too long or too short time windows are not conducive to driving behavior identification, 3.5 s being optimal. In addition, the relatively poorer identification of lane-change behaviors is attributed to the fact that lane-change behaviors are more complex.

In summary, the main contribution of this paper is to propose a real-time driving behavior identification framework based on the fusion of multi-source data, which innovatively integrates driver expressions into driving behavior identification. While driving, the driving behavior identification results are fed back to the driving monitoring platform in real time for driving style analysis and driving risk prediction, and can also be fed back to insurance companies for analysis, allowing them to provide more preferential policies for cautious and steady drivers. In addition, scores for drivers with respect to their driving behaviors can even be provided on a real-time basis to foster good driving habits.

In the next study, a more diverse sample of drivers will be employed (different driving skills, genders, and ages) to improve the generality of the research. Extra data sources could be considered, such as road alignment data, to further improve the identification results, and driving behavior categories can be refined. For example, turning behaviors can be further divided into left-turn and right-turn behaviors. In addition, the real-time identification can be upgraded to real-time prediction, so as to monitor driver states and predict driving risks timelier and more effectively.

## Figures and Tables

**Table 1:** Description of Vehicle Kinematic Data.

Field Name	Field Meaning	Unit	Min	Max
Time	Timestamp	s	-	-
Long	Longitude	°	118.679037	118.834188
Lat	Latitude	°	31.877423	31.987253
Velocity	Speed	km/h	0.00	113.25
Heading	Heading angle	°	0.00	359.99
Height	Height	m	0.00	60.78
Vert-vel	Vertical velocity	m/s	−4.57	4.79
Turn-radius	Turn radius	m	0.00	1000.00
AbsHead	Absolute heading angle	°	−2278.20	170.28
Longacc	Longitudinal acceleration	g	−29.66	1.68
Latacc	Lateral acceleration	g	−96.56	9.95
Rel-height	Elevation relative to the start point	m	−0.98	53.02

**Table 2:** Description of Action Units.

Action Unit	Description	Action Unit	Description
AU 01	Inner Brow Raiser	AU 15	Lip Corner Depressor
AU 02	Outer Brow Raiser	AU 17	Chin Raiser
AU 04	Brow Lower	AU 18	Lip Pucker
AU 05	Upper Lid Raiser	AU 20	Lip Stretcher
AU 06	Cheek Raider	AU 23	Lip Tightener
AU 07	Lid Tighter	AU 24	Lip Pressor
AU 09	Nose Wrinkler	AU 25	Lips Part
AU 10	Upper Lip Raiser	AU 26	Jaw Drop
AU 12	Lip Corner Puller	AU 27	Mouth Stretch
AU 14	Dimpler	AU 43	Eyes Closed

**Table 3:** Notation and description for variables used.

Symbol	Description	Unit
Longacc	The longitudinal acceleration	${m/s}^{2}$
$Longacc_{\max}$	The maximum longitudinal acceleration	${m/s}^{2}$
$Longacc_{\min}$	The minimum longitudinal acceleration	${m/s}^{2}$
yaw rate	The change rate of the heading angle	°/s
$t_{d}$	The duration	s
l	The length of the time window	s

**Table 4:** Optimal parameters of models.

Models	Main Parameters	Parameters Range	Optimal Parameters
S-LSTM	Number of units $N_{unit}$	$N_{unit}\in \left\{16,32,64,128\right\}$	$N_{unit}=32,\;m=128$ $D_{r}=0.2,\;R_{r}=0.2$ $l_{r}=10^{-3}$
	Batch size m	m∈{32,64,128,256}	
	Dropout rate $D_{r}$	$D_{r}\in \left\{0.1,0.2,0.5\right\}$	
	Recurrent dropout rate $R_{r}$	$R_{r}\in \{0.1,0.2,0.5\}$	
	Learning rate $l_{r}$	$l_{r}\in \left\{{\mathrm{10}}^{-3},{\mathrm{10}}^{-2}\right\}$	
ANN	Number of hidden layer units $N_{unit}$	$N_{unit}\in \left\{16,32,64,128\right\}$	$N_{unit}=64,\;l_{r}=10^{-3}$
	Learning rate $l_{r}$	$l_{r}\in \left\{{\mathrm{10}}^{-3},{\mathrm{10}}^{-2}\right\}$	
XGBoost	Maximum depth of each tree md	md∈{1,6,∞}	$md=6,\;l_{r}=0.3$
	Learning rate $l_{r}$	$l_{r}\in \left\{0.1,0.2,0.3,0.5\right\}$	

**Table 5:** Identification results of different input features.

Driving Behaviors	S-LSTM (ALL)			S-LSTM (VK)		
	Precision	Recall	F1	Precision	Recall	F1
Lane keeping	0.965	0.825	0.889	0.915	0.774	0.838
Acceleration	0.839	0.982	0.905	0.965	0.891	0.927
Deceleration	0.938	0.923	0.931	0.883	0.942	0.912
Turning	0.896	0.874	0.885	0.779	0.847	0.812
Lane change	0.753	0.798	0.775	0.640	0.750	0.691
Macro-average	0.878	0.880	0.877	0.837	0.841	0.836

**Table 6:** Identification results of different algorithms.

Driving Behaviors	ANN			XGBoost		
	Precision	Recall	F1	Precision	Recall	F1
Lane keeping	0.690	0.685	0.687	0.810	0.905	0.855
Acceleration	0.733	0.706	0.719	0.867	0.877	0.872
Deceleration	0.716	0.660	0.687	0.891	0.713	0.792
Turning	0.607	0.643	0.625	0.718	0.842	0.775
Lane change	0.517	0.570	0.542	0.689	0.716	0.702
Macro-average	0.653	0.653	0.652	0.795	0.811	0.799

## Data Availability

The naturalistic driving test data of this study are available from the corresponding author, upon reasonable request.
